# Designing a public engagement process for long-term urban park development project

**DOI:** 10.1371/journal.pone.0268804

**Published:** 2022-05-24

**Authors:** Myungjin Shin, Jung Hyun Woo, Hyeyoung Choi

**Affiliations:** 1 Interdisciplinary Program in Landscape Architecture, Graduate School of Environmental Studies, Seoul National University, Seoul, Republic of Korea; 2 City Form Lab, Department of Urban Studies and Planning, Massachusetts Institute of Technology, Cambridge, MA, United States of America; 3 School of Civil, Architectural Engineering and Landscape Architecture, Sungkyunkwan University, Suwon, Republic of Korea; Sri Eshwar College of Engineering, INDIA

## Abstract

Gathering public consensus about long-term urban open space development is more difficult than ever, even though public engagement is crucial for sustainable long-term policymaking. Routine evaluation of public awareness is important for retaining project momentum and designing appropriate public engagement processes for the future. This study focuses on the Yongsan Park Development Project, which has been in progress for more than three decades. An online survey of 2,000 respondents was conducted and analyzed to evaluate the current public awareness and ask questions about respondents’ expectations for public engagement. The results of this study reveal that 1) a hybrid methodology is needed to effectively approach different age groups; 2) an online survey can offer new insights for projects that repurpose U.S. army base and military sites into urban open spaces; 3) the survey results will enable us to design a better public participation process that is appropriate for post-pandemic society, in which virtual meetings and socially distanced communications are part of the new norm.

## 1. Introduction

Since the mid-20th century, there has been growing recognition of the importance of public engagement for a democratic society [1–4]. Community participation in particular has been emphasized for urban renewal and revitalization projects [5–7]. For example, as evidenced by the increase in the number of online survey sites and applications, public reception, or public engagement in general, is becoming a normalized procedure for research and public projects in South Korea [8, 9]. However, public engagement procedures for large-scale urban development and policymaking in a developed city can take a long time because many interconnected players are involved in the process. Furthermore, policy dictating a project is prone to change during the project development when multiple stakeholders are involved [10, 11]. Pressured by time constraint and growing demand for public engagement, as well as the current social distancing measures, survey is often adapted as a viable option.

The need to engage with a large population in a limited time is particularly pertinent during the development of urban open spaces because they not only have many stakeholders, including the foreign agencies such as the U.S. Forces Korea(USFK) and the U.S. Embassy, as well as Korean government sectors and agencies such as Ministry of Defense, Ministry of Land, Infrastructure, and Transportation(MOLIT), Cultural Heritage Administration, and the Seoul Metropolitan Government, just to list a few, but also affect the population at large when they are implemented. Such is the case for the Yongsan Park Development Project, a decades-long project to turn a former U.S. military garrison in South Korea into a large urban park upon the relocation of the army, followed by the return of the site to the Korean government. This project is unique in that it presents a new set of obstacles to designing an effective public engagement program. First, the complex stakeholder involvement means that public engagement program must be cleared of all bureaucratic hurdles. Because the project is closely related to the national defense agenda, it suffers from confidential limitations imposed on the public-access information. For example, in 2016, a judicial precedent kept environmental data about the site confidential until a media-induced public outcry forced the public access to the information [12]. Furthermore, the situation surrounding the project continues to shift, while little information is made public in advance. New areas continue to be selected for preopening, and recently, the U.S. Embassy decided to move to a location near the park site [13].

Due to the confidential nature of the military site, top-down communication and one-way information distribution by the government will continue despite the South Korean government’s continued efforts to increase the Yongsan Park Development Project’s public engagement programs. At the same time, however, urban planners, landscape architects, and policymakers need to understand the public’s current awareness levels and expectations so they can design an effective, appropriate public engagement program for the project despite the lack of public access to the site. Under the circumstances, surveys are required to understand the public opinion regarding the method and channels for adequate public communication and engagement.

To account for the diverse array of public engagement programs previously executed and to devise a more thorough plan, a comprehensive survey on the public awareness for the project is appropriate. In terms of methodology, online surveys are a viable research method for this study. The history of using surveys in the fields of public engagement and urban design [14–16] and the high rate of internet accessibility in South Korea, where some 99.7% of households have mobile access to the internet [17], strengthen the logic for using online surveys. In addition, internet-based communication between the government or institutions and individuals has been found to have significant credibility in South Korea [18].

Based on the conditions discussed above, we designed this study to answer three questions. The first question is about the public awareness of the Yongsan Park Development Project in South Korea. The second question explores the advantages of using online surveys as part of the public engagement program. We used the survey data to address our third question about developing an appropriate public engagement process for a long-term project at the national scale such as the Yongsan Park Development Project. Using our survey results as the medium, we further show that designing a public engagement program for long-term, large-scale urban open space development projects requires a heuristic approach based on routine evaluations of public awareness. Because government transparency and communication between the public and the government are crucial for building accountable and inclusive societies [3], we argue that devising an appropriate public engagement process for long-term urban projects contributes to sustainable development.

Finally, the Yongsan Park development project is important not only for its long history but also for its significance for other USFK sites. Along with the Busan Citizen Park, formerly Camp Hialeah, and civic park under development in Chuncheon City, previously Camp Page, the Yongsan Garrison function as examples for several USFK sites to be returned to the South Korean Government in the coming years [19]. Because the other sites are in similar situation to the Yongsan Garrison, the discussion presented here can serve as preliminary exploration regarding the future public engagement for many of such sites.

This study is organized as follows. First, a literature review of public engagement methods for urban projects is presented, followed by a review of studies on survey-based evaluation of public engagement in urban development. While this study traces the history of public engagement research to Arnstein’s model from 1969 [7], it also presents a number of criticisms and alternatives to public participation for urban development. Then, section on the research scope offers an in-depth explanation of Yongsan Park Development Project and the history of its public engagement programs. Methods section offers detailed explanation of the survey methodology and outcomes, including respondent statistics. Table 2, also included in this section, presents questionnaires with answer choices. Finally, results and the discussion are presented together in the following section. The results are clustered into four subsections which highlight the significance of this study. The final subsection of this chapter offers a public engagement model that can be adapted for long-term large-scale urban development projects to secure continued communication and discussion with the public. The findings are presented as a summary in the conclusion.

## 2. Literature review

### 2.1. Developments in public engagement methods for long-term, large-scale urban projects

Although a plethora of literature has examined public participation and urban design in general [15, 20–24], public engagement programs for urban open space development have yet to be fully discussed [25]. Some propose that in-person public engagement is a necessary step (for example, see Ostrom, 1998); however, the scale of national urban development projects such as Yongsan Park could become difficult hurdles for such an effort. Therefore, several types of remote public engagement methods should be used to buttress in-person methods.

Since the publication of the seminal article by Arnstein in 1969, Arnstein’s Ladder model has become the prototype for public participation in urban design studies [7]. Today, the main criticism faced by the Ladder model lies in the assumption that citizen participation is the goal instead of a tool or part of the process of building a policy [21, 26]. Because public engagement practice in the urban policy discourse has become normalized, Arnstein’s insistence on a hierarchical linear concept of public engagement in terms of power might now be insufficient. Furthermore, advances in technology and complicated, multi-party situations call for remote public engagement methods and the prioritization of communication [18, 22, 25].

Several public participation models have been proposed since Arnstein’s publication in 1969, but many of them still follow the Ladder concept [27, 28]. For example, Bobbio defines public participation as “a procedural tool which allows policymakers to include new actors in a policy network and entrust them with some design-related tasks” and posits three motivations for public participation in design-related policymaking: empowerment, legitimacy, and learning [25]. In laying out the different motives, he further notes that Arnstein’s model, while serving as a landmark in public participation discourse, has been criticized for its one-dimensionality that fails to address the diversity of public participation processes available today.

In fact, as far back as the 1980s, articles proposed to amend the limitations of the Arnstein model. In 1988, Connor proposed a systematic approach to the Ladder, eliminating the hierarchy and presenting a more integrated vision [29], and Davidson provided a cyclical model that avoided hierarchy entirely [30]. More recent models offer a framework that organizes different public engagement methods by policy status [31, 32].

Some researchers have noted that certain situations beyond government control require social learning rather than an exercise of citizen power [21]. Collins and Ison address that matter clearly in their attempt to create a new public participation paradigm, explaining that Arnstein’s model is insufficient to explain public engagement in overwhelmingly complicated issues such as climate change. They provide three critiques of the Arnstein model. First, as mentioned above, Arnstein postulates citizen control as the goal of participation, which can differ from a reality in which citizens participate for a diverse array of reasons other than control. Second, Arnstein posits a linear relationship between non-participation and citizen control, which fails to account for the uniqueness of contemporary policy problems. Third, Arnstein discussed public participation as a matter of power, but individuals participating in public engagement programs can have different interests that are typically flexible and subject to change over time. In line with others who discussed the limitations of the Arnstein model, Collins and Ison’s critique stems from the degree of abstraction in the model, which deviates dramatically from the realities of urban policymaking.

### 2.2. Survey-based evaluation of public engagement in urban development

One key issue that arose in our literature review is the need for a systematic evaluation of public engagement efforts. Maintaining and evaluating the level of public awareness throughout a project is particularly important for large, long-term projects. After an extensive review of the digital technologies available to public-engagement planners, Mandarano et al. criticized the descriptive tendencies of existing documents, which lacked an adequate level of evaluation [22]. The consensus is that public engagement efforts need to be evaluated to improve both the transparency and legitimacy of the process [33, 34].

In terms of evaluation methodology, several studies have offered insights. In their study about community-based planning for a participatory public open space in a Danish community, Pawlowski et al. distinguished between effect evaluation and process evaluation [24]. Effect evaluation is used to measure the effect of an intervention, whereas process evaluation describes process development and implementation. Huang’s survey-based study of a citywide park policy is a typical effect evaluation; its aim is to evaluate a previously implemented urban park development policy [15]. Although public engagement efforts about urban planning and related policymaking have mostly focused on effect evaluations [15, 24, 33], a few programs have focused on the design or evaluation of the process [21, 35].

In terms of survey methodology, remote surveys are a growing market that can deliver data previously unavailable to researchers. Because in-person discussions are believed to be effective and efficient, many people working in the public engagement sector have prioritized in-person dialogues over remote options [36]. An alternative can be found in the Yokohama project, which transformed a former military base into a public park. Its participatory design process focused on communicating and collecting information through five layers of participation: competition, public voting, public promotion, exhibitions and symposiums, and continued information distribution [35]. Because the Yokohama case required public awareness, its public engagement managers focused on consistency in the distribution of communication. However, the Yokohama model is appropriate for its specific context; if one was to apply it to the Yongsan Park Project, it would need to expand the public engagement to national level. Also, because the project took place prior to the COVID-19 pandemic, it did not account for the social distancing measures. Since social distancing is key to dealing with the pandemic, now is a good time to consider using public engagement alternatives such as remote surveys and virtual meetings to replace in-person participation, as well as to search for new public engagement processes. Online survey platforms offer specific options to cater to researchers in these situations. For example, Open Survey, an online survey agency in Korea, maintains a pool of participants who receive benefits and incentives for their responses and provides researchers with clean data analyses [37]. For statewide surveys, such as those needed for the Yongsan Park development, online surveys also serve as efficient alternative to in-person options because they incur no delays in receiving answers from multiple respondents at once. In order to continue public engagement during urban open space planning while considering the changing social conditions, a comprehensive public engagement program that includes procedures for the collection of public opinion regarding not only the project but the evaluation of public engagement program is necessary.

## 3. Research area: The Yongsan Park development project and its history of public engagement

**Fig 1:**
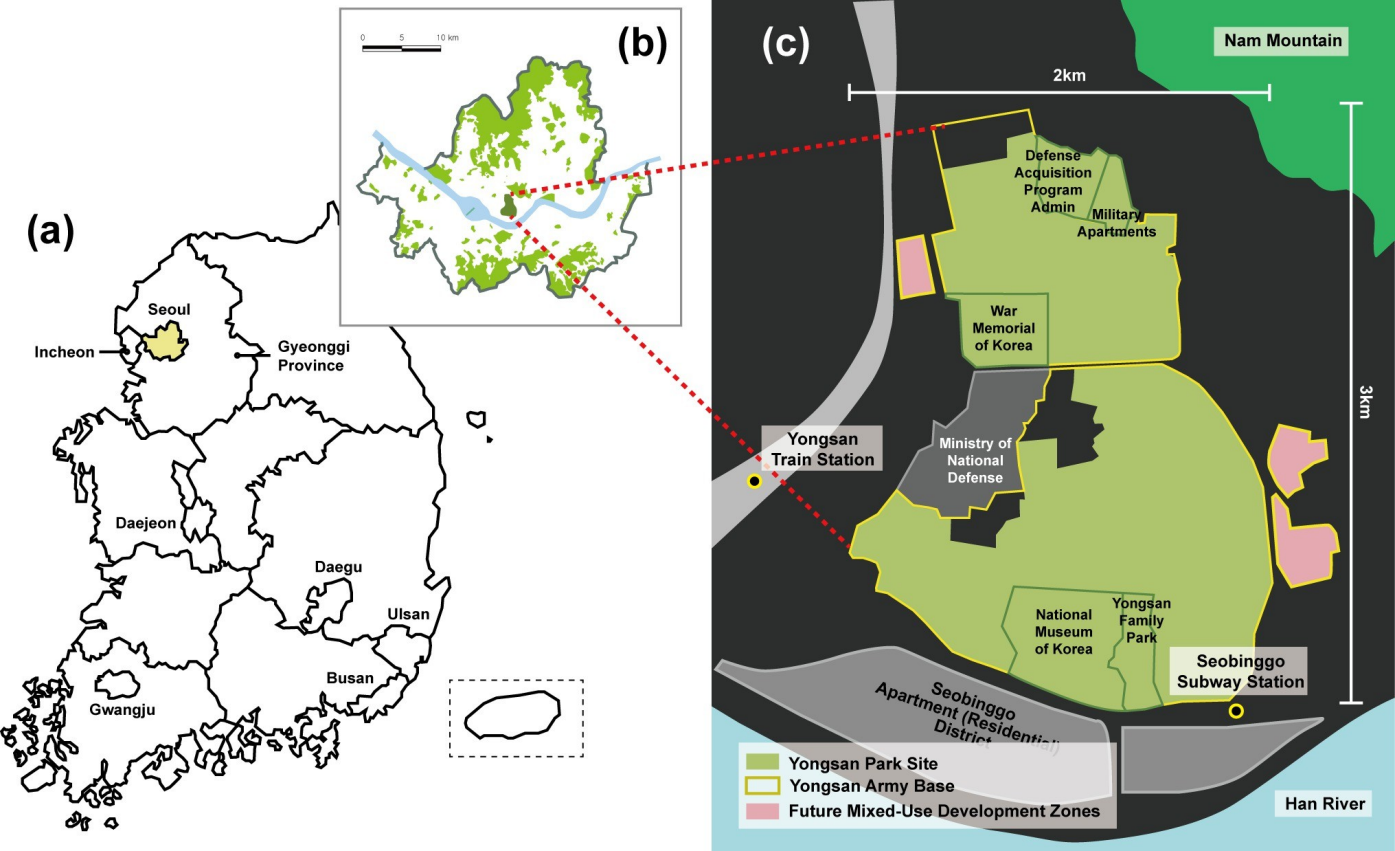
Yongsan Park development project location. (a) Map of South Korea (b) Location of the Yongsan Park site in Seoul (c) Yongsan Park site.

**Fig 2:**
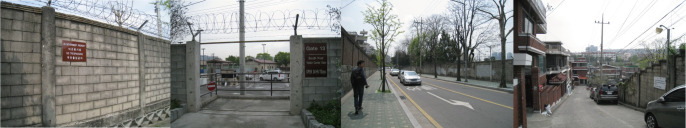
Views of the Yongsan Park boundary.

Yongsan Park is a large-scale project whose purpose is to develop a national urban park in South Korea, which will be located in the center of Seoul (Figs 1 and 2). Heralded as the first national urban park, the conditions surrounding the unprecedented urban open space development process and the multi-layered stakeholders, ranging from foreign governments to military to citizen brigades, often hinder a rapid development process.

The site, spanning more than 300ha, has a century-long history of foreign military occupation since the Qing military occupied the area in the late 19^th^ Century [38]. In 1945, the U.S. Army replaced the Japanese Imperial army, thereby commencing the U.S. Army Occupation era of the site. Although discussions about returning the military-occupied land to the Korean Government and subsequent development of the site as a park began as early as 1988 under President Roh, Tae-woo, it took almost three decades for the park’s master plan to be finalized [39, 40].

The project was designed as two-stage process: the relocation of the foreign military base to the city of Pyeongtaek, and the subsequent transformation of the post-industrial military base into an urban public park. During the early years, the project proceeded in a top-down manner since most of the information were confidential military and diplomatic matters between South Korea and the United States. The complex situation of the Yongsan Garrison did not allow for public engagement beyond information distribution by the government. In fact, much of the information regarding the Yongsan Garrison remains confidential and undisclosed to the public for security reasons. Because of such condition, the project remained largely hidden from the public eye despite its scale.

In the mid-90s, however, public engagement practice became more normalized in South Korea. Although the top-down approach to public relations for Yongsan Park development remained, the government and public organizations made several attempts to engage the public in more diverse ways. Public hearings and on/offline surveys were the most typical options for the public to deliberate their opinions. Between 1996 and 2019, 28 public events, including public discussions, seminars, forums, symposiums, and roundtables were held. Most were hosted by the state or local government [41], with only one third of them organized by civic groups [39] and no guarantee that the public’s opinion would be adopted at all. In addition, between 1990 and 2019, ten separate on/offline surveys were conducted to understand public opinion about the project (Table 1).

**Table 1:** On/offline surveys about Yongsan Park development by year.

Year	1990	2004	2006	2009	2010	2015	2018–2019
Number of Offline Surveys	1	2	1	1	2	0	0
Number of Online Surveys	0	0	0	0	1	2	1

Data reconstructed from [39].

More recent public engagement projects for the Yongsan Park development, such as the 2009 Yongsan Park Idea Competition, the 2013 Open Workshop for Yongsan Park Basic Design, and the 2018 Yongsan Garrison Bus Tours, testify to the government’s new interest in public engagement. With more than 2,600 participants overall, the bus tour demonstrates significant interest in providing opportunities for the public to engage with the project [39, 42]. However, even after these events, the public were not given an opportunity to deploy their ideas and experience into the park design, as the Master Plan was already under way. Thus, the top-down public engagement programs continued while the demand for raised level of participation increased. The online survey presented in this study and the subsequent public engagement program design, both executed with the support of MOLIT, may be described as a response to such demand.

## 4. Methods

### 4.1. Survey method

This research used an online survey panel, with nation-wide 2,000 responses collected via survey agency’s mobile application. The survey period was October 26^th^ and 27^th^, 2020, and the questions comprised eleven single selections and two multiple selections in Korean. A member of the authors for this paper was involved in devising the survey questions as part of the MOLIT consultants as seen in Table 2.

**Table 2:** Survey questions and answer choices.

Type	Question No.	Question	Answer Choices
Project Awareness	Q1	Are you aware of what the Yongsan Garrison site will become in the future?	1. Yes, I know what it will become. 2. No, I do not know what it will become.
Project Awareness	Q2	Please specify what the Yongsan Garrison site will become in the future.	1. Park 2. Museum 3. Theme Park 4. Residential Area (Apartment Complex) 5. Commercial Space 6. Other
Preferred Communication Channel	Q3	If you answered YES to question 1, how did you become aware of that information?	1. Newspaper/broadcast 2. Internet/social network 3. Yongsan Park Development Project website/official social network page 4. Promotional materials (posters, brochure, etc.) 5. Acquaintances and friends 6. Other
Project Awareness	Q4	Some parts of the Yongsan Garrison (near Seobingo Subway Station) have been open to public access since August 2020. Were you previously aware of this information?	1. Yes, I was aware of this information. 2. No, I was not aware of this information.
Preferred Communication Channel	Q5	If you answered YES to question 4, how did you become aware of that information?	1. Newspaper/broadcast 2. Internet/social network 3. Yongsan Park Development Project website/official social network page 4. Promotional materials (posters, brochure, etc.) 5. Acquaintances and friends 6. Other
Park Development Issues	Q6	To return the Yongsan Garrison site to the Korean public, the Yongsan Park Development Project has been under way. As the largest-ever national urban project in Seoul center, this project is expected to require many resources and a long time. What do you believe is the most pertinent opinion that must be reflected in the project? Please select up to two (2) options from below.	1. All four (4) of the options below 2. Long-term vision of the government 3. Expert advice 4. Opinions of residents living near the park site 5. Opinions from the general public 6. Other
Preferred Communication Channel	Q7	How should the Yongsan Park development process and related information be communicated to the Korean public? Please select up to two (2) options from below.	1. Newsletter service 2. Periodic brochures (reports) 3. Social networks/media (blog, social networks, etc.) 4. Direct communication (public hearings, etc.) 5. Public-run media platform 6. Other
Public Participation	Q8	The following questions ask for your thoughts on public participation in the Yongsan Park Development Project. Please answer the following questions under the assumption that these public participation projects will take place.	1. (Go next question.) 2. (No answer required)
Public Participation	Q9	Who should a public participation group contain? Please select the best option from below.	1. Anyone interested in park development 2. Those living near the park development site 3. Those with some expertise in park development 4. NGOs and citizen groups 5. Other
Public Participation	Q10	Which generation should lead the public participation for the Yongsan Park Development Project?	1. People in their 60s and older 2. People in their 40s and 50s 3. People in their 20s and 30s 4. Other
Public Participation	Q11	How should public discussion topics be selected?	1. Topics selected by experts 2. Topics selected by the public participation group 3. Topics selected by experts and the public participation group 4. Other
Public Participation	Q12	If you were to join the public participation group for the Yongsan Park Development Project, what topics would you like to discuss?	1. Yongsan Park identity and meaningLocal community and Yongsan ParkEcological/cultural/historical understanding of the parkHistorical/cultural heritage of the Yongsan Park vicinity 2. Other
Public Participation	Q13	The public participation group for the Yongsan Park Development project is expected to meet for six (6) months. After the participation program is finished, how should public engagement for this project continue?	1. Constantly via a website 2. Through the existing public participation group or the selection of a new group 3. Periodic public hearings and debates 4. Community service or historical and cultural programs onsite

The Open Survey company in South Korea, an online survey company that secures one of the largest open mobile research panels for effective random sampling, conducted the entire survey on behalf of the authors and MOLIT. The survey used a sampling error of ±1.44% with at an 80% significance level. The mathematical formula for the sampling error is shown in the following Eq (1):

$$E=1.28\times \sqrt{\frac{p(1-p)}{n}}$$ (1)

- E = sampling error
- p = observed probability
- n = number of samples

### 4.2. Survey data

The maximum observed probability (p in the equation) was set to 0.5. The survey participants can be categorized into groups by age, gender, occupation, and household geographic information. All participants (n = 2,000) were Korean nationals, where 1,000(50%) participants were female and the other 1,000(50%) male. The ages ranged between 14 to 69, with the average of 39.5 years (SD = 15.85). We categorized them into six age groups: teenagers (14–19), 20s, 30s, 40s, 50s, and 60s and older. Highest percentage of respondents were from Seoul in terms of geographic information (31%), and office workers had the highest response rate (53.8%). The descriptive statistics were summarized using SPSS v.25 and are shown in Table 3.

**Table 3:** Descriptive statistics of the respondents.

Category	Subcategory	Percentage (%)	Average Age (Years)	Standard Deviation (Years)	Number of Responses
Age	Teenagers (14–19)	16.6			332
	20s	16.7			334
	30s	16.7			334
	40s	16.7			334
	50s	16.7			334
	60s and older	16.6			332
	Total (14-60s and older)	100	39.5	15.85	2,000
Gender	Women	50			1,000
	Men	50%			1,000
Occupation	Middle/High school student	10.5			210
	College/Graduate student	9.8			195
	Office worker	53.8			1,075
	Housewife	13.2			263
	Other (self-employment, salesperson, independent contractor, and farmer)	12.8			257
Household Geographic Information	Seoul	31			622
	Gyeonggi Province and Incheon (Seoul Metropolitan Area)	34.3			685
	Five Metropolitan Cities (Busan, Daegu, Gwangju, Daejeon, Ulsan)	17.1			342
	Other	17.5			351

The survey contained thirteen questions in three areas to examine (1) public awareness about the Yongsan Park project, (2) effective communication channels, and (3) ideal roles for those selected to develop the park. This survey is a significant part of the overall public engagement process to prepare for the next phase of public engagement and public participation. Overall, the results showed that different age groups demonstrated varied understanding of the project as well as preferences for public engagement process. The entire result table for the survey is shown in S1 Table.

## 5. Results and discussion

### 5.1. Limited public awareness

Questions Q1, Q2, and Q4 asked about respondents’ opinion on and awareness of the future use and current development stage of the park project (Table 4). In question 1, only 571 (28.5%) respondents replied that they knew what was planned for the site. Of the 571 respondents, only 319 (55.9%) correctly answered question 2 by indicating that the Yongsan Garrison site will be turned into a park following the relocation of the army. Question 4 asked about the recent opening of a limited area near the Seobinggo subway station area at the Yongsan Park site (Fig 1). Only 537 (26.9%) of the 2,000 respondents were aware of this public event.

**Table 4:** Survey results regarding the public awareness of the park project.

Question	Answer Choice	Total	Age					
			Teenager (14–19)	20s	30s	40s	50s	60s and older
1	1	28.5% (571)	11.1% (37)	18.0% (60)	29.0% (97)	28.4% (95)	39.8% (133)	44.9% (149)
	2	71.5% (1429)	88.9% (295)	82.0% (274)	71.0% (237)	71.6% (239)	60.2% (201)	55.1% (183)
	Total	100.0% (2000)	100.0% (332)	100.0% (334)	100.0% (334)	100.0% (334)	100.0% (334)	100.0% (332)
2	1	55.9% (319)	45.9% (17)	41.7% (25)	57.7% (56)	57.9% (55)	55.6% (74)	61.7% (92)
	2	3.7% (21)	10.8% (4)	6.7% (4)	4.1% (4)	2.1% (2)	2.3% (3)	2.7% (4)
	3	7.7% (44)	5.4% (2)	5.0% (3)	6.2% (6)	8.4% (8)	8.3% (11)	9.4% (14)
	4	25.9% (148)	21.6% (8)	33.3% (20)	24.7% (24)	26.3% (25)	29.3% (39)	21.5% (32)
	5	6.8% (39)	16.2% (6)	13.3% (8)	7.2% (7)	5.3% (5)	4.5% (6)	4.7% (7)
	Total	100.0% (571)	100.0% (37)	100.0% (60)	100.0% (97)	100.0% (95)	100.0% (133)	100.0% (149)
4	1	26.9% (537)	11.7% (39)	16.8% (56)	24.6% (82)	30.5% (102)	38.0% (127)	39.5% (131)
	2	73.2% (1463)	88.3% (293)	83.2% (278)	75.4% (252)	69.5% (232)	62.0% (207)	60.5% (201)
	Total	100.0% (2000)	100.0% (332)	100.0% (334)	100.0% (334)	100.0% (334)	100.0% (334)	100.0% (332)

Respondents in their 50s and 60s showed higher awareness of the park project, 55.6% and 61.7%, respectively, whereas people in their teens and 20s were less aware, 45.9% and 41.7%, accounting for 37 and 60 respondents, respectively. The reason for that difference is likely the prolonged project duration, which gave the older generation more chances to be exposed to the news. Those in their teens and 20s were born during or after the early 2000s, after the park development project had taken off; thus, unless they encountered the news or had a specific interest in the urban parks, they had little chance to become aware of this long-term project.

Despite the public events, forums, symposiums, gallery exhibitions, and bus tours into the U.S. garrison conducted during the past few decades, our survey results indicate that the public remains largely unaware of the project. This may be problematic since the project is forecasted to be left unfinished for at least another decade into the future. As the political debates regarding the land use in Yongsan district that are the results of increased demand for housing in Seoul continue to rise, one finds a number of attempts by politicians and lobbying groups criticizing the park development [43]. Once the discussion was made the news, the public demand for termination of such efforts followed. Since Yongsan Park development will remain in progress for several years to come, public advocacy is crucial for successful completion of the project. Therefore, it is necessary to devise a public engagement method that allows the public to become aware of the park and gather correct information during the rest of this long-term project. As mentioned in the previous section, public engagement during urban open space planning that responds to the changing social conditions, a comprehensive public engagement program that includes procedures for the collection of public opinion. With the project schedule that continues to be delayed, use of digital technologies for routine evaluation of public awareness level for the project is necessary. A method to raise project awareness among the younger generation in a way that will inspire a long-term interest in the project is necessary.

Furthermore, the regional analysis as demonstrated in Table 5 shows that those living in Seoul had the greatest project awareness, with 237 respondents (41.5%) having correct information, followed by the metropolitan area outside Seoul, with 109 respondents (19.1%). Because the Yongsan Park Development Project is funded by the national budget, public opinion from every corner of the nation is as important as the opinion of those residing in or around Seoul. However, our survey results show that only those living in or near the site were aware of the project. It is important to devise a plan that can raise public awareness throughout the nation, as the Yongsan Park Development Project is a national project–in other words, it will be built using national government funding.

**Table 5:** Survey results regarding the public awareness of the park project (Household geographic information).

Question No.	Answer Choice	Total	Household Geographic Information			
			Seoul	Gyeonggi Province and Incheon in Seoul Metropolitan Area	The Five Metropolitan Cities (Busan, Daegu, Gwangju, Daejeon, Ulsan)	Others
Q1	1	28.5% (571)	38.1% (237)	27.0% (185)	22.5% (77)	20.5% (72)
	2	71.5% (1429)	61.9% (385)	73.0% (500)	77.5% (265)	79.5% (279)
	Total	100.0% (2000)	100.0% (622)	100.0% (685)	100.0% (342)	100.0% (351)
Q2	1	55.9% (319)	57.0% (135)	58.9% (109)	50.6% (39)	50.0% (36)
	2	3.7% (21)	3.8% (9)	3.8% (7)	1.3% (1)	5.6% (4)
	3	7.7% (44)	6.3% (15)	9.2% (17)	10.4% (8)	5.6% (4)
	4	25.9% (148)	27.0% (64)	20.0% (37)	32.5% (25)	30.6% (22)
	5	6.8% (39)	5.9% (14)	8.1% (15)	5.2% (4)	8.3% (6)
	Total	100.0% (571)	100.0% (237)	100.0% (185)	100.0% (77)	100.0% (72)
Q4	1	26.9% (537)	31.0% (193)	27.4% (188)	21.3% (73)	23.6% (83)
	2	73.2% (1463)	69.0% (429)	72.6% (497)	78.7% (269)	76.4% (268)
	Total	100.0% (2000)	100.0% (622)	100.0% (685)	100.0% (342)	100.0% (351)

### 5.2. Effective communication channels

Questions Q3, Q5, and Q7 asked respondents about their preferred communication channels for information delivery and the methods by which they prefer to share information to support the next phase of public engagement and participation (Table 6). Newspapers and broadcast news are the main media through which 203 (63.6%) of the 319 respondents to question 3 learned that the Yongsan site will become a park. Approximately 64% of respondents in their 30s and older knew about the Yongsan Park project through newspaper and broadcast news channels, whereas only 35% of people in their teens and 20s knew about the project. Among all respondents 24.8% learned about the project from the internet and SNS channels: 47.1% of teenagers, 32% of people in their 20s, and approximately 20% of people in their 30s and older.

**Table 6:** Survey results regarding the preferred communication channels by age.

Question No.	Answer Choice	Total	Age					
			Teenager (14–19)	20s	30s	40s	50s	60s and older
Q3	1	63.6% (203)	35.3% (6)	36.0% (9)	64.3% (36)	72.7% (40)	64.9% (48)	69.6% (64)
	2	24.8% (79)	47.1% (8)	32.0% (8)	25.0% (14)	21.8% (12)	24.3% (18)	20.7% (19)
	3	0.9% (3)	-	-	-	-	1.4% (1)	2.2% (2)
	4	1.6% (5)	-	4.0% (1)	1.8% (1)	-	2.7% (2)	1.1% (1)
	5	8.5% (27)	17.6% (3)	24.0% (6)	8.9% (5)	5.5% (3)	5.4% (4)	6.5% (6)
	6	0.6% (2)	-	4.0% (1)	-	-	1.4% (1)	-
	Total	100.0% (319)	100.0% (17)	100.0% (25)	100.0% (56)	100.0% (55)	100.0% (74)	100.0% (92)
Q5	1	58.7% (315)	25.6% (10)	32.1% (18)	59.8% (49)	69.6% (71)	64.6% (82)	64.9% (85)
	2	22.7% (122)	41.0% (16)	32.1% (18)	22.0% (18)	16.7% (17)	18.9% (24)	22.1% (29)
	3	3.9% (21)	5.1% (2)	7.1% (4)	2.4% (2)	2.9% (3)	3.1% (4)	4.6% (6)
	4	1.1% (6)	5.1% (2)	3.6% (2)	-	1.0% (1)	0.8% (1)	-
	5	12.5% (67)	23.1% (9)	21.4% (12)	12.2% (10)	8.8% (9)	12.6% (16)	8.4% (11)
	6	1.1% (6)	-	3.6% (2)	3.7% (3)	1.0% (1)	-	-
	Total	100.0% (537)	100.0% (39)	100.0% (56)	100.0% (82)	100.0% (102)	100.0% (127)	100.0% (131)
Q7	1	19.4% (388)	19.3% (64)	16.5% (55)	16.5% (55)	18.0% (60)	19.8% (66)	26.5% (88)
	2	9.5% (190)	10.8% (36)	8.7% (29)	12.0% (40)	9.3% (31)	9.0% (30)	7.2% (24)
	3	55.8% (1116)	73.8% (245)	68.6% (229)	61.1% (204)	53.0% (177)	41.9% (140)	36.4% (121)
	4	62.6% (1253)	46.4% (154)	58.4% (195)	59.9% (200)	65.6% (219)	74.3% (248)	71.4% (237)
	5	32.5% (649)	27.1% (90)	29.9% (100)	31.7% (106)	34.4% (115)	33.8% (113)	37.7% (125)
	6	0.2% (4)	-	0.3% (1)	0.3% (1)	-	0.3% (1)	0.3% (1)
	Total	180.0% (3600)	177.4% (589)	182.3% (609)	181.4% (606)	180.2% (602)	179.0% (598)	179.5% (596)

*Q7 is a multiple-selection question, which resulted in the cumulative percentage above 100%.

Question 5 asked how respondents learned about the recent opening event. Overall, 537 respondents were aware of the opening. Among those in their 30s and older, 60–70% learned about it from newspapers and broadcast news, but only 20–30% of those in their teens and 20s who knew about the opening learned about from those channels. On the other hand, 30–40% of those in their teens and 20s learned the news from the internet and SNS channels, whereas only 15–20% of people in their 30s and older learned about it that way.

Question 7 asked how respondents would prefer to receive information about the progress in developing Yongsan Park. 62.7% of the 2,000 respondents said they prefer public hearings; 55.8% preferred social media such as blogs and SNS, and 32.5% would like to learn from reporters and articles. By age, those in their teens, 20s, and 30s preferred social media over public hearings.

The medium through which respondents came to know about the park development project differed by generation. In all cases, the most popular information channels were newspaper and traditional news media or the internet and social networks. However, people in their teens and 20s came to know about the project mainly through the internet and social networks, whereas people ages 30 and older came to know about the project mainly via newspapers and news broadcasts. The relocation of the U.S. military is a necessary precursor to project continuation. However, the relocation process remains confidential due to its relevance to military and diplomatic relationships between the U.S. and Republic of Korea, not to mention the international politics surrounding the USFK. Although the public is aware that several USFK sites are planned to be returned to the Korean government, the exact time and method for each site remains confidential. Therefore, information about it has often been communicated only through official press releases or traditional news media, which helps explain why public awareness about the project was significantly lower among those in their teens and 20s than among those age 30 and older.

One interesting result is that 3 (17.6%) of the teens and 6 (24%) of the people in their 20s responded in question 3 that they learned about the project from acquaintances. Although those numbers are not proportionately significant, they likely learned about the project from parents, grandparents, schoolteachers, or professors who had been exposed to the news longer and learned about the project through various media.

However, with the COVID-19 pandemic expected to make lasting impact on the field of public participation with the increased use of digital technology across the generations [44], in combination with the high internet accessibility in South Korea [18], one can expect that using internet-based communication method will not be a significant deterrent in the future. Based on this information and the need to increase the public awareness among the younger generations, the official information regarding Yongsan Park should focus on internet-based communication methods in the future.

### 5.3. Devising a national public participation program

Finally, the questions Q6 and Q9–Q13 asked respondents to describe their ideal role as members of the public and propose an effective strategy for park developers who will form the core of public engagement and participation in Yongsan Park development as public opinion representatives (Table 7). These questions were designed to help devise a follow-up public engagement process.

**Table 7:** Survey results regarding the public engagement strategy.

Question No.	Answer Choice	Total	Age					
			Teenager (14–19)	20s	30s	40s	50s	60s and older
Q6	1	23.3% (465)	22.0% (73)	23.7% (79)	24.6% (82)	26.3% (88)	22.8% (76)	20.2% (67)
	2	20.0% (400)	9.3% (31)	17.1% (57)	23.7% (79)	24.6% (82)	23.1% (77)	22.3% (74)
	3	36.7% (734)	35.2% (117)	36.2% (121)	33.2% (111)	32.0% (107)	37.7% (126)	45.8% (152)
	4	38.4% (767)	54.2% (180)	44.0% (147)	38.3% (128)	34.1% (114)	28.7% (96)	30.7% (102)
	5	45.1% (903)	43.1% (143)	44.0% (147)	42.5% (142)	44.6% (149)	50.3% (168)	46.4% (154)
	6	0.4% (8)	0.3% (1)	0.6% (2)	0.6% (2)	-	0.6% (2)	0.3% (1)
	Total*	163.8% (3277)	164.2% (545)	165.6% (553)	162.9% (544)	161.7% (540)	163.2% (545)	165.7% (550)
Q9	1	53.4% (1069)	49.4% (164)	50.9% (170)	56.0% (187)	60.5% (202)	53.0% (177)	50.9% (169)
	2	16.7% (333)	25.3% (84)	23.4% (78)	16.2% (54)	12.6% (42)	12.0% (40)	10.5% (35)
	3	17.8% (356)	13.9% (46)	17.7% (59)	15.6% (52)	12.9% (43)	22.5% (75)	24.4% (81)
	4	11.7% (234)	11.4% (38)	7.8% (26)	11.7% (39)	13.8% (46)	12.0% (40)	13.6% (45)
	5	0.4% (8)	-	0.3% (1)	0.6% (2)	0.3% (1)	0.6% (2)	0.6% (2)
	Total	100.0% (2000)	100.0% (332)	100.0% (334)	100.0% (334)	100.0% (334)	100.0% (334)	100.0% (332)
Q10	1	1.8% (37)	0.6% (2)	0.9% (3)	1.2% (4)	0.3% (1)	1.5% (5)	6.6% (22)
	2	45.0% (899)	25.3% (84)	26.3% (88)	31.4% (105)	59.0% (197)	64.4% (215)	63.3% (210)
	3	44.0% (879)	68.7% (228)	64.7% (216)	57.8% (193)	31.1% (104)	24.0% (80)	17.5% (58)
	4	9.3% (185)	5.4% (18)	8.1% (27)	9.6% (32)	9.6% (32)	10.2% (34)	12.7% (42)
	Total	100.0% (2000)	100.0% (332)	100.0% (334)	100.0% (334)	100.0% (334)	100.0% (334)	100.0% (332)
Q11	1	11.2% (224)	10.2% (34)	9.9% (33)	14.1% (47)	11.1% (37)	9.0% (30)	13.0% (43)
	2	19.1% (382)	26.2% (87)	20.4% (68)	17.1% (57)	19.5% (65)	17.7% (59)	13.9% (46)
	3	69.5% (1391)	63.0% (209)	69.8% (233)	68.6% (229)	69.5% (232)	73.4% (245)	73.2% (243)
	4	0.1% (3)	0.6% (2)	-	0.3% (1)	-	-	-
	Total	100.0% (2000)	100.0% (332)	100.0% (334)	100.0% (334)	100.0% (334)	100.0% (334)	100.0% (332)
Q12	1	13.8% (275)	23.2% (77)	18.0% (60)	15.3% (51)	10.8% (36)	8.7% (29)	6.6% (22)
	2	20.3% (405)	25.0% (83)	27.8% (93)	20.1% (67)	18.6% (62)	14.1% (47)	16.0% (53)
	3	48.1% (962)	38.3% (127)	38.0% (127)	47.9% (160)	50.0% (167)	59.3% (198)	55.1% (183)
	4	17.5% (350)	13.3% (44)	15.3% (51)	16.2% (54)	20.7% (69)	17.7% (59)	22.0% (73)
	5	0.4% (8)	0.3% (1)	0.9% (3)	0.6% (2)	-	0.3% (1)	0.3% (1)
	Total	100.0% (2000)	100.0% (332)	100.0% (334)	100.0% (334)	100.0% (334)	100.0% (334)	100.0% (332)
Q13	1	29.9% (599)	43.7% (145)	32.0% (107)	29.6% (99)	27.5% (92)	25.4% (85)	21.4% (71)
	2	11.5% (230)	19.0% (63)	12.6% (42)	10.8% (36)	8.4% (28)	9.0% (30)	9.3% (31)
	3	38.3% (765)	22.3% (74)	31.4% (105)	36.5% (122)	42.8% (143)	49.4% (165)	47.0% (156)
	4	20.0% (399)	14.8% (49)	22.8% (76)	22.5% (75)	21.3% (71)	16.2% (54)	22.3% (74)
	5	0.4% (7)	0.3% (1)	1.2% (4)	0.6% (2)	-	-	-
	Total	100.0% (2000)	100.0% (332)	100.0% (334)	100.0% (334)	100.0% (334)	100.0% (334)	100.0% (332)

*Q6 is a multiple-selection question, which resulted in the total answer above 100%.

Question 9 asked about the characteristics and composition of park development leaders. 53.5% of the 2,000 respondents answered that a leader can be anyone interested in park development. 17.8% of the 2,000 respondents said only professional groups working as park designers and planners can be leaders. Question 10 asked which age group would make ideal leaders for the park development. The answers to this question revealed that each age group demonstrated affinity towards their own age groups. In other words, those in their teens, 20s, and 30s preferred the leaders to be in their 20s–30s, whereas the respondents in their 40s, 50s, and 60s answered that people in their 40s–50s will make ideal public leaders.

This finding supports the previous criticisms against Arnstein’s public participation model. As discussed by Collins and Ison, the heavily abstracted Ladder model as presented by Arnstein fails to represent the complicated realities of urban policymaking [21]. This set of questions asked for the public opinion regarding the public participation process in terms of public representation; unlike the Ladder model, our finding demonstrates that there may be a different viewpoints and agenda within the participating public, which may or may not consider increase of citizen power as the goal in public participation [7].

In fact, the disparity among the public regarding the goal of public participation is visible in question 11 and 12. 69.6% of the 2,000 respondents to question 11 showed that professionals and leaders should develop and determine discussion topics. In question 12, the respondents were asked about the discussions they would like to participate in should they become part of the park development leaders. 48.1% of the 2,000 participants wanted to discuss the ecological, cultural, and historical elements of the park for future park programs. After completing their time as a leader, 38.3% of the participants said they would prefer to attend regular public hearings and public participation events to provide public opinion.

Based on our survey results and analysis, MOLIT devised a public participation program, titled the National Public Participation Group (NPPG), in late 2020. The purpose of the program was to allow people who would like to be part of the Yongsan Park Development Project to discuss and create a public proposal by the end of July 2021. The resulting proposal is planned to be adapted in the revision of the Yongsan Park Master Plan. During the past 30 years of park development, the project has been subjected to continued criticism about the lack of substantial public participation [39]. In this sense, the NPPG is the first significant public engagement specifically planned for the Yongsan Park Development Project.

The results of our survey in relation to public participation are as follows. First, the results showed that about half of the NPPG should be those in their 20s and 30s, and the other half should be in their 40s and 50s. Second, when asked about the issues that should be discussed regarding park development in question 6, most respondents agreed that the opinion of ‘the public at large’ is necessary (903 responses, 45.1%); however, a significant number answered that local residents’ commentary (767 responses, 38.4%) and expert opinions (734 responses, 36.7%) should also have some weight. Third, most respondents (1,391, 69.6%) agreed in question 11 that the topics to be discussed during public participation should be determined by both the public and experts. Fourth, the preferences for information communication channels during park development, given in answer to question 7, were direct communication (1,253, 62.6%), online communication (1,116, 55.8%), and public-participation media platforms (649, 32.5%). Finally, according to the answers to question 12, the most popular discussion topics were ecological, cultural, and historical programs (962 responses, 48.1%), followed by park development and the local community (405 responses, 20.3%) and Yongsan Park’s historical and cultural heritage (350 responses, 17.5%).

The results demonstrate that the older generation tended to give expert opinions and the government’s attitude greater importance, whereas the younger generation tended to see public opinion as more important. The different age groups also preferred different communication methods; whereas the older generation preferred direct communication, the younger generation leaned toward social networks. Thus, according to the survey, diverse array of communication channels is necessary when designing a NPPG that caters to a diverse spectrum of age groups. This finding aligns with the results of several studies on public engagement, which found that a hybrid methodology is preferred by both respondents and researchers to account for generational and regional differences [5, 25, 33].

**Fig 3:**
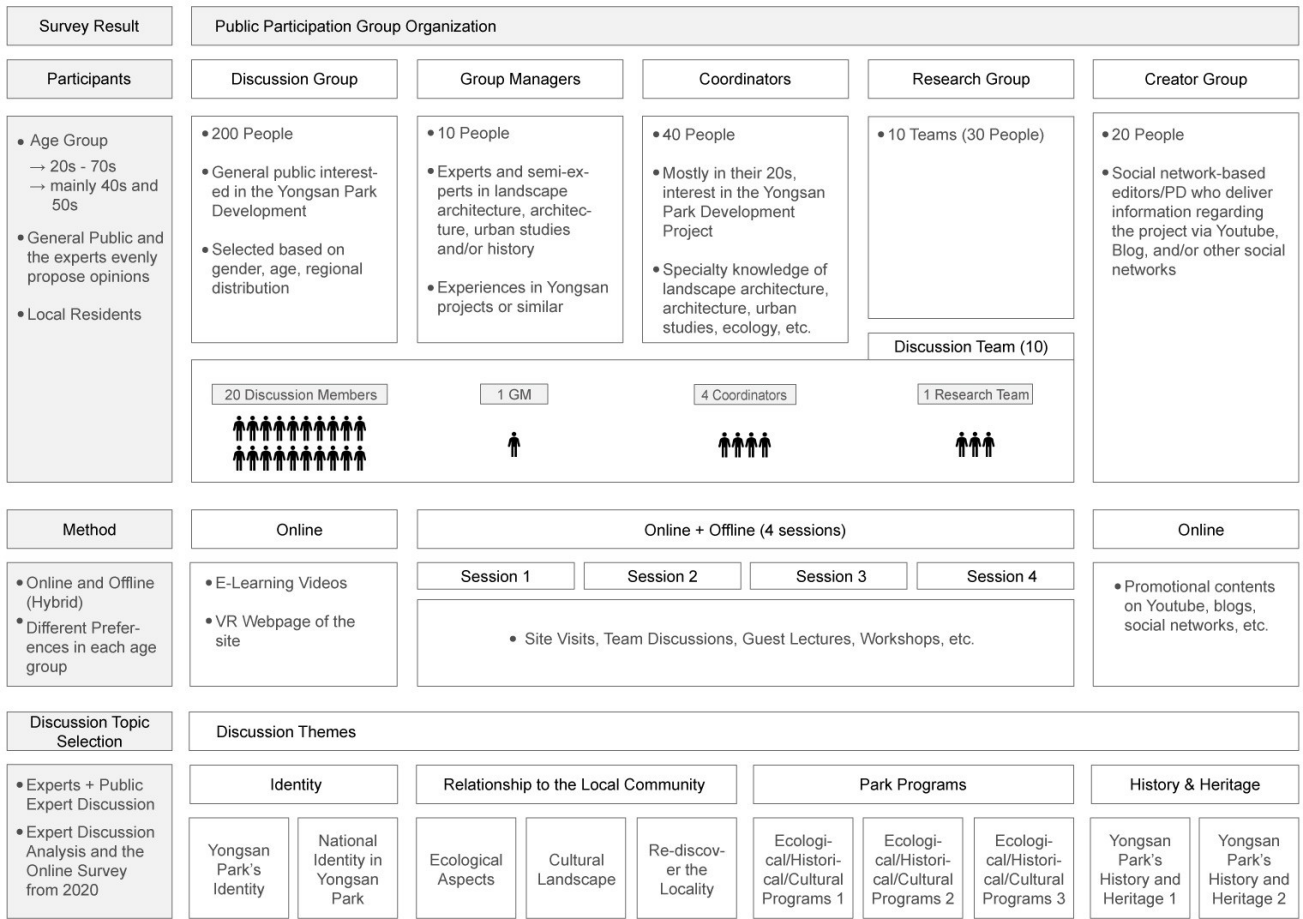
Organization of the national public participation group (2021).

**Fig 4:**
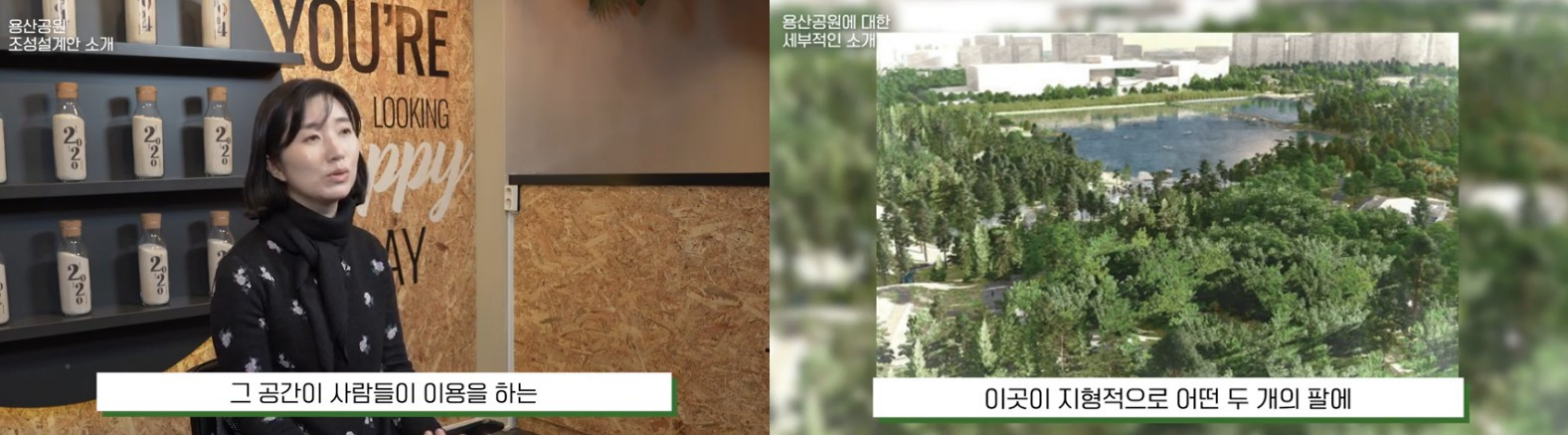
National public participation group online education. source: authors.

**Fig 5:**
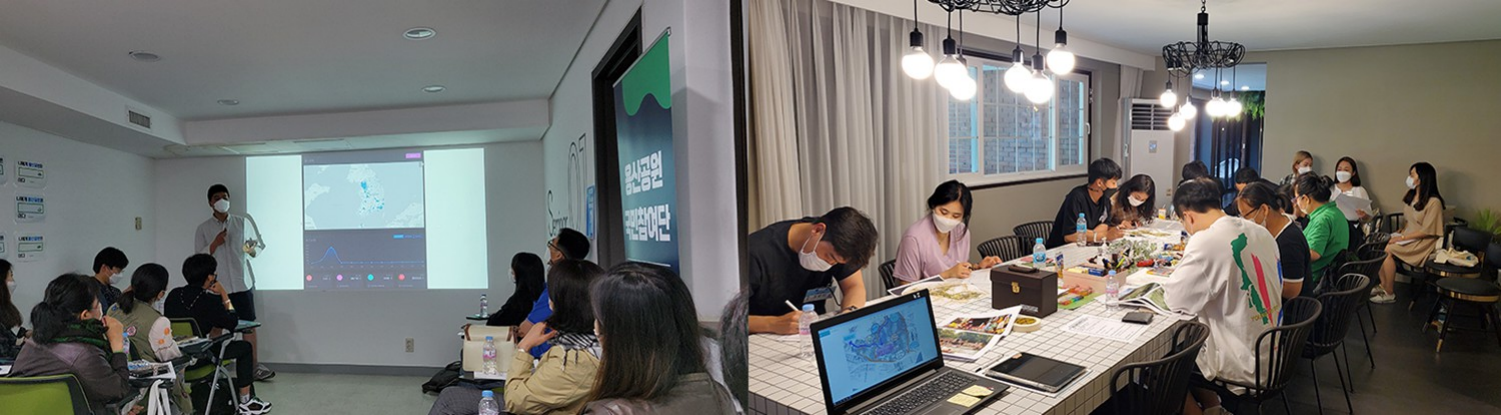
National public participation group meetings. source: authors.

Based on the result analysis, the NPPG was designed and organized to cater to people interested in the park development while maintaining diversity in gender, age, residence, and expertise regarding landscape design. Most of the NPPG members had tertiary education in landscape, architecture, and ecology, but some come from diverse backgrounds such as film, performance, and law. Instead of randomly selecting members of the public, the NPPG members were selected through double screening process, including in-person interviews. Considering the pandemic, the program was designed to meet the social distancing measures. For example, the NPPG program was designed to incorporate both online and offline components. Although the structure of the topics discussed by the NPPG were extracted from previous research [10], the number of discussion teams was determined based on the survey results from this study (Figs 3–5).

### 5.4. Usability of the online survey tool in large-scale projects

As discussed in the literature review, online survey platforms can be a viable option for collecting public opinion regarding a large-scale project over a long period of time. Today, online survey platforms also offer a mobile option. A key advantage that a mobile-based online survey has over traditional methods is that allows seamless collection of each respondent’s locational data. This way, the survey can take place over a large area with controlled respondent sample. In case of the Yongsan Park Development Project, which needs opinions from Koreans residing in all corners of the nation, this function proves to be useful. Although online surveys do not employ multi-dimensional survey method at the moment, they are a viable way to acquire immediate answers to questions regarding public awareness or opinions.

**Fig 6:**
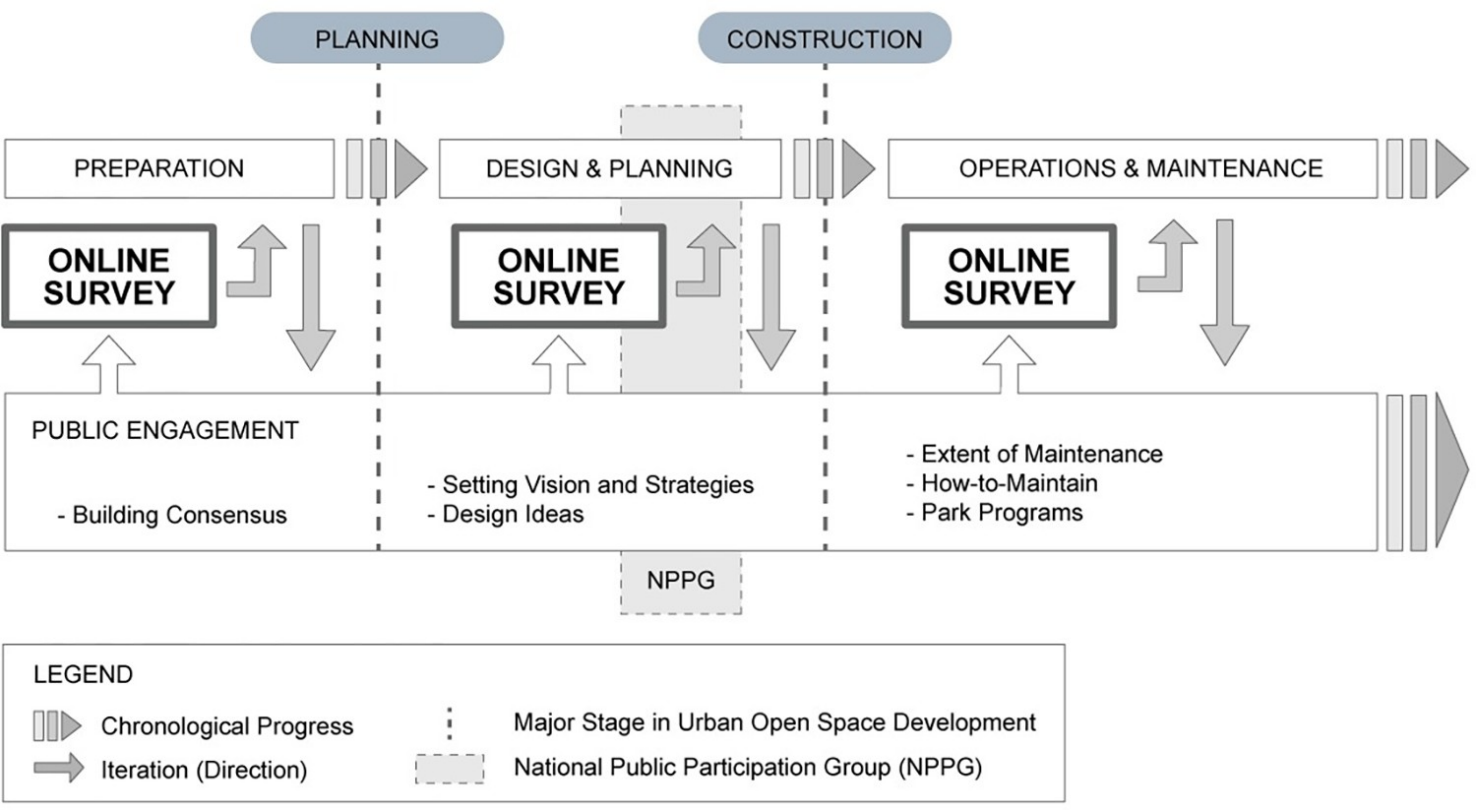
Adoption of online surveys in public engagement processes for developing urban open spaces.

Furthermore, online surveys are useful at every step of a long-term development project, from preparation through planning and design to public engagement, construction, and management. Understanding the public awareness, preferences, and needs of different regional, age, and occupation groups is necessary in determining the public relations tasks of each planning and design phase. The results of such surveys might inspire ideas about park management and public programs. Updated knowledge about what the public needs in terms of participation methods and communication media will be useful in designing public engagement programs. Therefore, surveys and similar methods should not be singular events during the overall public engagement process but rather form an iterated series of public programs, as shown in our proposed Public Engagement Process Model (Fig 6).

In our model, public engagement does not consist of a specific hierarchy of public participation, as in Arnstein’s Ladder model [7]. Instead, public engagement is a continuous effort that reflects the changing situation of developing urban open spaces. Because the Ladder model’s goal is to increase citizen control, it is difficult to apply to urban development whose goal is to change the urban landscape through citizen empowerment; with different agendas from each stakeholder group vying for different results, even among the public, the public participation process should be open-ended with flexible and heuristic characteristics. In fact, based on the results of this study, we propose that routine and continued evaluation of the public participation process by the public will serve as an important threshold in maintaining the flexible quality in the public engagement.

Thus, our model was inspired by the Ladder model but uses a series of online surveys to mediate between the public engagement and urban development processes. Public awareness and engagement expectations are constantly evaluated through the surveys, which allow urban development project managers to adjust the public engagement process accordingly. Instead of formulating a rigid framework of public engagement, our model allows for technological shortcuts that enable heuristic approaches to designing and implementing public engagement programs.

## 6. Conclusion

With the successful termination of NPPG in July 2021, followed by a series of public engagement programs, this study offers the following valuable points. First, a mobile-based online survey determined public awareness about the Yongsan Park Development Project and found that most were unaware of the project despite decades-long public engagement efforts. Second, we explored the advantage of using mobile-based online surveys as part of the process. As shown in Fig 4, routine adoption of online surveys throughout the development process is useful. Finally, we designed a public engagement process. Because the target public is broad and diverse, a hybrid methodology combining online, and offline public engagement techniques is necessary. Although the public engagement process model proposed in this study is inspired by Arnstein’s idea of public participation, public engagement process is useful only when it is constantly evaluated through routine evaluations.

In addition, the example of the Yongsan Park Development Project is significant as it serves as precursor to many of the USFK garrison sites that will be returned to the Korean Government in the future. Because the complex international relationship surrounding the project is unlikely to be resolved in near future, it will be important to devise public engagement process that takes advantage of the possible public engagement tools at hand with utmost efficiency.

Despite those strengths, the interpretation of our study results should be limited to confirming a general tendency of public opinion because comparable pre-existing data do not exist. Should we continue to evaluate public awareness of the project using online surveys in the future, the shifts in public awareness in accordance to the program may be plotted. Such empirical inquiry into the NPPG will help us determine the efficacy of the current public engagement program for the Yongsan Park Development Project.

## Supporting information

S1 Table Yongsan Park development project online survey results (2020). (XLSX)

## References

[1] Callahan K. Citizen Participation: Models and Methods. International Journal of Public Administration. 2007;30: 1179–1196.

[2] Michels A, De Graaf L. Examining Citizen Participation: Local Participatry Policy Making and Democracy. Local Government Studies. 2010;36: 477–491.

[3] Nations United. Transforming Our World: The 2030 Agenda for Sustainable Development. United Nations; 2015. Report No.: A/RES/70/1.

[4] OECD. Recommendation of the Council on Open Government. OECD/LEGAL/0438; 2021.

[5] Roberts N. Public deliberation in an age of direct citizen participation. The American Review of Public Administration. 2004;34: 315–353. doi: 10.1177/0275074004269288

[6] Linn K. Building Commons and Community. Oakland, CA: New Village Press; 2007.

[7] Arnstein SR. A Ladder of Citizen Participation. Journal of the American Planning Association. 2019;85: 24–34. doi: 10.1080/01944363.2018.1559388

[8] Kim YJ. An Assessment of the Digital Gaming Platforms’ Viability. Master’s Thesis, Ewha Women’s University. 2013.

[9] Hahn S, Yoon J-H, Kim J-M. Extending the Technology Acceptance Model to Examine the Intention to Use Tourism Applications on Smartphone. Korean Journal of Hospitality and Tourism. 2014;23: 19–40.

[10] MoLIT. Yongsan Park Development Plan In-Depth Analysis and Public Paricipation Content Development. Seoul: MoLIT; 2020.

[11] Learning from Nodeul Island [Roundtable]. Space. Feb 2020: 66–75.

[12] Jang SW, Park KJ, Lee HS. Cancellation of the Denial to the Information Disclosure regarding the Environmental Data from the U.S. Yongsan Garrison. Seoul; 2016 19. Report No.: 2015구합72610.

[13] Park J-Y. Yongsan Garrison Sportsfield and Softball Field to Pre-Open this Year. In: ChosunBiz [Internet]. 14 May 2021 [cited 25 Jun 2021]. Available: https://biz.chosun.com/policy/policy_sub/2021/05/16/SH5HNQRYXJFPNKEK4PEI3ZRV5U/.

[14] Saroff JR, Levitan AZ. Survey Manual for Comprehensive Urban Planning. Anchorage, Alaska: Development Research Associates, Inc.; 1969. Report No.: 19.

[15] Huang S-CL. The Impact of Public Participation on the Effectiveness of, and Users’ Attachment to, Urban Neighbourhood Parks. Landscape Research. 2010;35: 551–562. doi: 10.1080/01426397.2010.504916

[16] Djukić A, Marić J, Antonić B, Kovač V, Joković J, Dinkić N. The Evaluation of Urban Renewal Waterfront Development: The Case of the Sava Riverfront in Belgrade, Serbia. 2020; 17.

[17] Ministry of Science and ICT, National Information Society Agency. Summary of the 2020 National Survey on the Internet Use. Seoul: National Information Society; 2020.

[18] Hong H. The Internet, transparency, and government–public relationships in Seoul, South Korea. Public Relations Review. 2014;40: 500–502. doi: 10.1016/j.pubrev.2014.01.011

[19] USFK Returns Land to South Korea 주한미군, 대한민국으로 미군기지 반환. In: United States Forces Korea [Internet]. [cited 15 Nov 2021]. Available: https://www.usfk.mil/Media/Press-Releases/Article/2037248/usfk-returns-land-to-south-korea/.

[20] Hanzl M. Information technology as a tool for public participation in urban planning: a review of experiments and potentials. Design Studies. 2007;28: 289–307.

[21] Collins K, Ison R. Jumping off Arnstein’s ladder: social learning as a new policy paradigm for climate change adaptation. Env Pol Gov. 2009;19: 358–373. doi: 10.1002/eet.523

[22] Mandarano L, Meenar M, Steins C. Building Social Capital in the Digital Age of Civic Engagement. Journal of Planning Literature. 2010;25: 123–135. doi: 10.1177/0885412210394102

[23] Seltzer E, Mahmoudi D. Citizen Participation, Open Innovation, and Crowdsourcing: Challenges and Opportunities for Planning. Journal of Planning Literature. 2013;28: 3–18. doi: 10.1177/0885412212469112

[24] Pawlowski CS, Winge L, Carroll S, Schmidt T, Wagner AM, Nørtoft KPJ, et al. Move the Neighbourhood: Study design of a community-based participatory public open space intervention in a Danish deprived neighbourhood to promote active living. BMC Public Health. 2017;17: 481. doi: 10.1186/s12889-017-4423-4 28526028 PMC5438546

[25] Bobbio L. Designing effective public participation. Policy and Society. 2019;38: 41–57. doi: 10.1080/14494035.2018.1511193

[26] Painter M. Participation and Power. Citizen participation in government. Sydney: Hale & Ironmonger; 1992. pp. 21–36.

[27] International Association for Public Participation. IAP2 Spectrum of Public Participation. In: IAP2 [Internet]. 2019 [cited 14 May 2021]. Available: https://www.iap2.org.au/resources/iap2-published-resources/.

[28] Kim J, Kim S. Citizen Participation in Digital Public Administration. Journal of Korean social welfare administration. 2021;23: 175–205.

[29] Connor DM. A New Ladder of Citizen Participation. National Civic Review. 1988;77: 249–257.

[30] Davidson S. Spinning the wheel of empowerment. Planning. 1998;1262: 14–15.

[31] Warburton D, Wilson R, Rainbow E. Making a Difference: A guide to evaluating public participation in central government. London: Department for Consistutional Affairs; 2007.

[32] CAPIRE. The Engagement Triangle. CAPIRE; 2015. Available: https://capire.com.au/communities/publications/.

[33] Griffin GP, Jiao J. Crowdsourcing Bike Share Station Locations. Journal of the American Planning Association. 2019;85: 35–48. doi: 10.1080/01944363.2018.1476174 PMC11139253 38817633

[34] Kim JY. Speculative Development and Publicness: The Case of Yongsan Area. Journal of Korean Urban Geographical Society. 2016;19: 29–41.

[35] Park J-H, Son Y-H, Tsuge K. Participatory Design Process for the Utilization of the Military Relocation Site—The Case of the Idea Competition for the Fukaya Communication site in Yokohama. Journal of the Korean Institute of Landscape Architecture. 2011;39: 10–25.

[36] Ostrom E. A Behavioral Approach to the Rational Choice Theory of Collective Action. The American Political Science Review. 1998;92: 1–22.

[37] Choi JW. Who will own the rising mobile research market? Excellence Marketing for Customer. 2015;49: 62–70.

[38] Choi J-H, Nam Y-W. The Heritage Value of Yongsan Military Base and its Direction of Urban Development. Journal of the Korean Urban Geographical Society. 2014;17: 1–13.

[39] Choi H. The Development Process of Yongsan Park and its Resilience. Ph.D Dissertation, Seoul National University. 2020.

[40] Chira S, Times ST the NY. In Heart of Seoul, an Unwanted U.S. Presence (Published 1988). The New York Times. 14 Aug 1988. Available: https://www.nytimes.com/1988/08/14/world/in-heart-of-seoul-an-unwanted-us-presence.html. Accessed 8 Oct 2020.

[41] MoLIT. Yongsan Park Roundtable 1.0. Seoul: MoLIT; 2017.

[42] MoLIT. Yongsan Garrison Bus Tour to Continue in 2020. MoLIT; 2019.

[43] Kang S. Looking at Yongsan Park as the government fails to find new housing development site… initiates discussion over 80 thousand public housing on the park site [집 지을 땅 못찾자 용산공원 손댄 與..공공주택 8만호 추진]. Edaily. 4 Aug 2021. Available: https://www.edaily.co.kr/news/read?newsId=03381686629143712&mediaCodeNo=257. Accessed 15 Mar 2022.

[44] How COVID-19 triggered the digital and e-commerce turning point. In: UNCTAD [Internet]. [cited 29 Mar 2022]. Available: https://unctad.org/news/how-covid-19-triggered-digital-and-e-commerce-turning-point.

